# Accuracy of the Apple Watch 4 to Measure Heart Rate in Patients With Atrial Fibrillation

**DOI:** 10.1109/JTEHM.2019.2950397

**Published:** 2019-12-13

**Authors:** Dhruv R. Seshadri, Barb Bittel, Dalton Browsky, Penny Houghtaling, Colin K. Drummond, Milind Desai, A. Marc Gillinov

**Affiliations:** 1 Department of Biomedical EngineeringCase Western Reserve University 2546 ClevelandOH44106USA; 2 Heart and Vascular InstituteCleveland ClinicClevelandOH44106USA

**Keywords:** Wearable sensors, Apple Watch, heart rate, cardiac arrhythmias, atrial fibrillation, accuracy, clinical trial

## Abstract

Background Wearable wrist-monitors offer an unobtrusive way to acquire heart rate data in an efficient manner. Previous work in this field has focused on studying healthy subjects during exercise but has yet to assess the efficacy of these devices in patients suffering from common cardiac arrhythmias such as atrial fibrillation. Objective The objective of this pilot study was to assess the accuracy of the Apple Watch heart rate monitor in fifty patients experiencing atrial fibrillation compared to telemetry. Results Results from this pilot clinical study demonstrated a correlation coefficient of 0.7 between all readings on the Apple Watch and telemetry. Furthermore, the Apple Watch assessed heart rate more accurately in patients who were in atrial fibrillation than in those that were not (r_c_ = 0.86, patients in AF, vs. r_c_ = 0.64, patients not in AF). Clinical Impact The presented data from this pilot study suggests that caution should be noted before using the Apple Watch 4 wearable wrist monitor to monitor heart rate in patients with cardiac arrhythmias such as atrial fibrillation.

## Graphical abstract

Apple Watch Series 4 includes a built in ECG sensor to assess heart rhythm and detects heart rate via photoplethysmography (PPG). A green light source (~540 nm) illuminates the skin and a photodetector measures the intensity of the reflected light. Heart rate is then estimated based on the pulsatile changes in reflected light induced by fluctuations in blood flow every heartbeat.

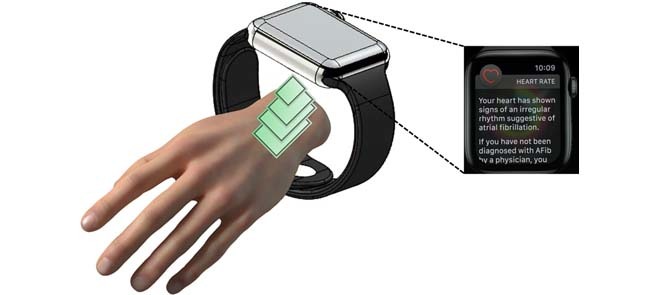

## I. Introduction

Atrial fibrillation mymargin and atrial flutter (AF, collectively) are the most common types of cardiac arrhythmia and affect over five million people in the United States [1]. Recent studies have suggested that ~700,000 people in the United States may have previously undiagnosed AF, thus, highlighting the need for the development and validation of accurate, non-invasive technologies to enable patients to track when they might be experiencing AF [2], [3]. The technological maturation and validity of wearable devices has recently been questioned by the medical community due to the lack of clinical data assessing their accuracy and efficacy in the identification and monitoring of human diseases [4]. Authors of this study and others have assessed the accuracy of wrist-worn monitors to measure heart rate in healthy subjects [5]–[8]; however, there is limited research in such devices to accurately monitor heart rate in individuals with cardiac arrhythmias such as AF [9]–[11]. The recent media attention surrounding the diagnosis of AF with the FDA-cleared ECG sensor on the Apple Watch (AW4) raised an important question: how reliable and accurate is this specific device in measuring heart rate in patients with AF? Building off of this hype, this study assessed the accuracy of the AW4 HR monitor function in patients with AF to determine whether or not the AW4 should be used in the clinic.

## II. Study Design and Human Subject Protocol

### A. Number of Subjects and Recruitment

Fifty post-operative cardiac surgery patients admitted to the cardiac telemetry step-down floors at the Cleveland Clinic Main Campus from January 2019 to March 2019 were recruited by trained research personnel within five days post-surgery. All patients gave written informed consent to participate in the study. The protocol was approved by the Cleveland Clinic institutional review board (IRB) and registered at clinicaltrials.gov (NCT03798613). The study lasted from December 21st, 2018 till March 29th, 2019. The mean (SD) age of the patients was 61.4 ± 10.4years; the mean ± st. dev body mass index (calculated as weight in kilograms divided by height in meters squared) was 30.4 ± 4.87; 14 participants were women (28%), and 3 participants were African American (6%). All patients were on continuous six-lead telemetry according to standard clinical practice on the step-down floor. 50% of subjects experiencing atrial fibrillation and 50% experiencing sinus rhythm at the time of enrollment were studied.

### B. Inclusion and Exclusion Criteria

Inclusion criteria included patients above the age of 18 years and post-operative cardiac surgery patients on the cardiac telemetry floors. Exclusion criteria included patients with a cardiac pacemaker, use of a radial artery graft for coronary artery bypass grafting (CABG), known chronic or persistent heart rhythm disorders, and tattoos located on the skin of the wrist or forearm where the AW was placed.

### C. Confidentiality and Safety Protocols

Data was inputted and analyzed from REDCAP by trained research personnel who were a part of the IRB protocol. The authors take responsibility for the data presentation and analysis in this study.

## III. Methods and Procedures

### A. Data Collection

Five AW’s and five iPhone 8’s was purchased. The right and left wrist circumference of each patient was measured prior to placing the AW. Each patient was outfitted with an AW for no more than five minutes and the location (left or right wrist) was randomly assigned via a randomization grid. All devices were thoroughly and antiseptically cleaned by the research staff according to hospital guidelines before and after each patient’s testing. Each enrolled patient had a minimum of three assessments of heart rate per day for at least two days, generating a minimum of six data points per patient.

### B. Data Analysis

All statistical analyses were performed using R and SAS statistical software (SAS v9.4; SAS, Inc., Cary, NC). Graphics were constructed using SAS.

## IV. Results and Clinical Outcome Analysis

Across the five watches and iPhone 8’s purchased, 266 heart rate values were recorded. Twenty-four data points were not obtained. Missing data can be attributed to the following reasons. For one subject, none of the five AW’s was able to read an ECG rhythm at all six time points; the same watches were fine for the other subjects. In two other subjects, there was missing data due to the inability to obtain both AW and telemetry readings at certain time points due to early patient discharge. Lastly, one subject withdrew consent from study participation.

**FIGURE 1.**
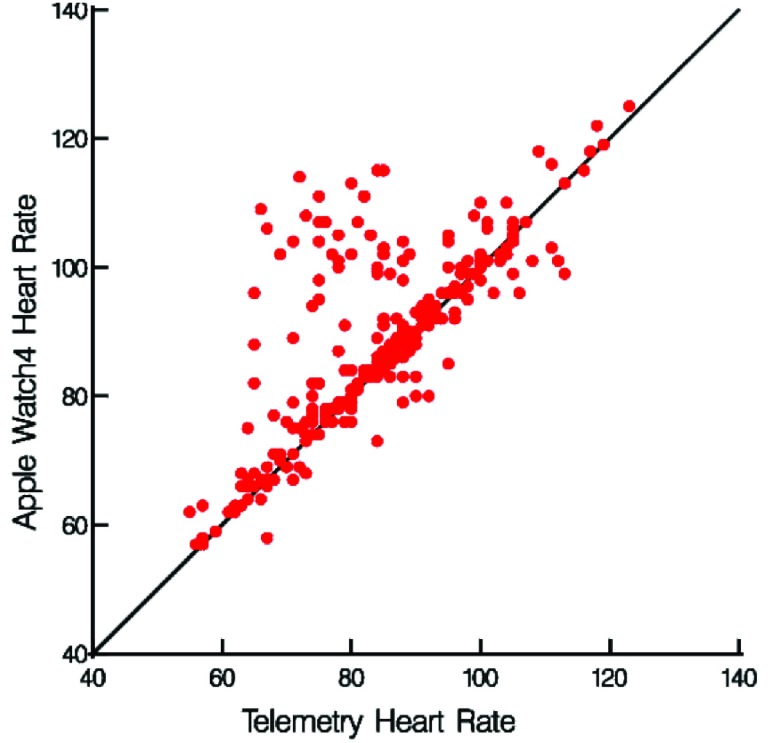
Heart rate measurements from telemetry and the Apple Watch 4.

Heart rate ranges for the AW and telemetry ranged from 57 – 125 bpm (mean 88 ± 14) and 54 – 137 (mean 86 ± 15) respectively. Lin’s concordance correlation coefficients (r_c_) were calculated to provide a measure of agreement for the AW with telemetry. The concordance correlation coefficient (CCC) measures the degree to which the paired observations fall on the identity line. Overall there was an agreement of r_c_ = 0.7 between the AW and telemetry (0.63, 0.75, 95% confidence interval), (**Figure 1**).

**FIGURE 2.**
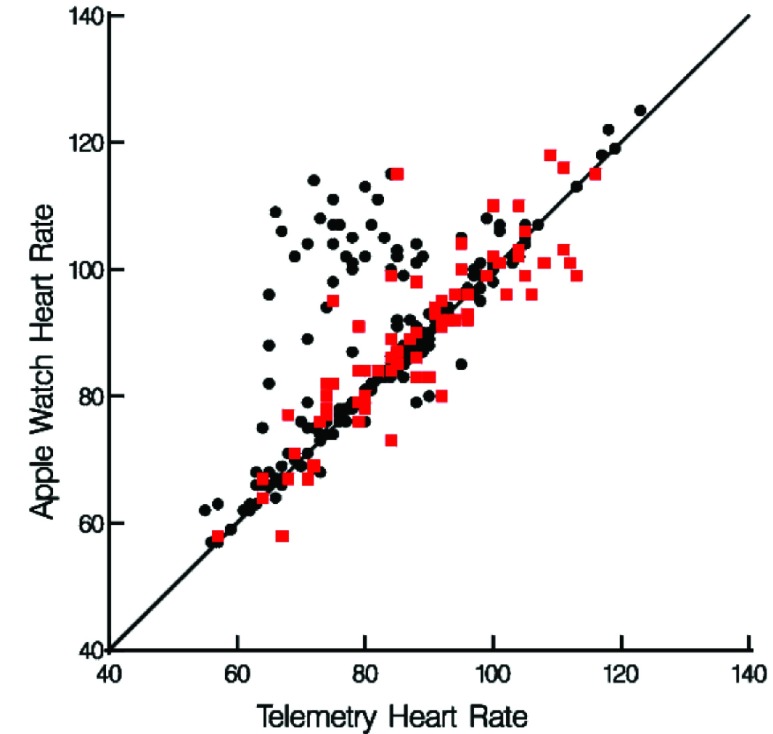
Heart rate measurements from telemetry and the Apple Watch 4 stratified by atrial fibrillation on telemetry (red = Afib, black = no Afib).

The AW assessed HR more accurately in patients who were in AF than in those that were not (r_c_ = 0.86, patients in AF, vs. r_c_ = 0.64, patient not in AF) (**Figures 2** **and** **3**).

**FIGURE 3.**
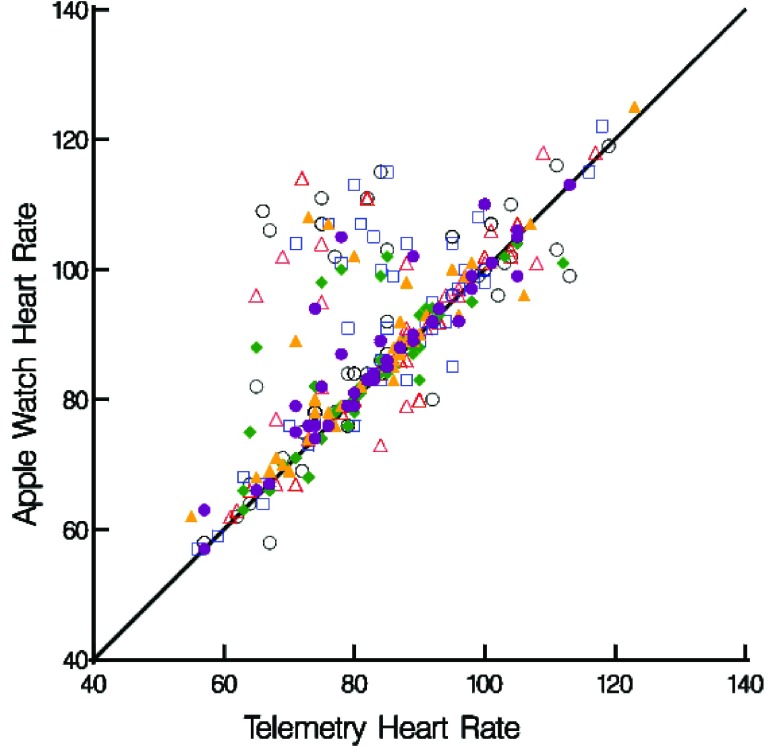
Heart rate measurements from Telemetry and Apple Watch4 stratified by Time point (Day 1 measures 1–3 (open shapes), Day 2 measures 4–6 (filled shapes)).

**FIGURE 4.**
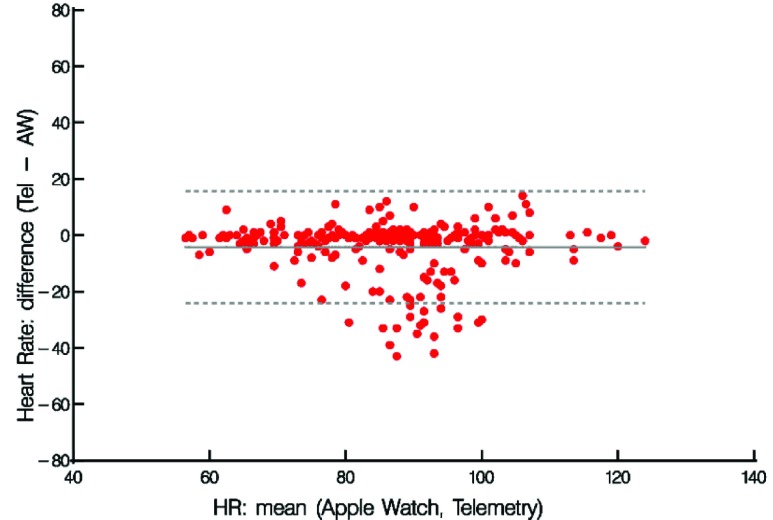
Heart rate measurements from Telemetry and Apple Watch 4 stratified by Time point (Day 1 measures1–3 (open shapes), Day 2 measures 4–6 (filled shapes)).

Paired differences were calculated by subtracting the measured heart rate (HR) from the heart rate recorded on the Telemetry under each condition and at each time point. Summary data are provided for relative differences, absolute differences, and percent differences ((HRtel – HRwatch)/ HRtel). Bland-Altman analysis was performed and data was plotted to assess agreement against the mean values (**Figure 4**). The motivation to plot using this method was to uncover any tendency for the variation to change with the magnitude of the measurement. Results suggest quite a bit of disagreement between the mean values thereby highlighting potential inaccuracies with the AW4 HR sensor compared to measurements from telemetry.

Furthermore, the study investigated whether the subjects with lower HR correlations might have had other arrhythmias, or might have had other sources of noise in the ECG traces (**Table 1**).

**TABLE 1:** Association Between Heart Rate Correlations and Arrhythmias as Adjudicated by a Cardiologist

HR Difference Range	Rhythm from Telemetry	Rhythm from AW4
0–10	Ventricular Ectopy	Ventricular Ectopy
	None	Ventricular Ectopy
	Atrial Ectopy	Atrial Ectopy
	Noise	Ventricular Ectopy
11–20	Atrial Ectopy	Sinus
	None	Ventricular Ectopy
	Ventricular Ectopy	Ventricular Ectopy
	Sinus	Ventricular Ectopy
21–30	Sinus	Atrial Ectopy
31–40	Ventricular Ectopy	Noise

The overall goal of this pilot study was to assess the accuracy of the AW4 in measuring heart rate compared to telemetry in patients in the cardiac step-down unit following surgery. Our pilot study currently represents the first such clinical study evaluating the efficacy of this particular device to measure heart rate in a controlled clinical environment. The r_c_ value of 0.7 was lower than those determined from prior studies by members of this team. Wang et al. evaluated the efficacy of the AW 3 (and other wrist-monitors) compared to the Polar H7 monitor in 50 healthy subjects and found that the AWs had a r_c_ value of 0.91 [5]. Etiwy et al. assessed the accuracy of the AW 3 (and other heart rate monitors) compared to standard ECG limb leads and the Polar H7 ECG chest strap monitor in 80 patients with established cardiovascular disease enrolled in phase II or III cardiac rehabilitation (CR) [8]. The study found that overall the agreement between the AW and ECG was (r_c_ = 0.8). Our results coupled with those published by members of our team suggest the need for subsequent validation studies in the clinic to further elucidate such differences which could have enormous ramifications if heart rate readings from these devices are used to inform clinical decisions in patients with cardiac arrhythmias.

The difference in heart rate among patients with AF and sinus or other rhythms presented the following question: did the patient know they were in AF and therefore may have been more precise at getting the watch reading? A key learning from our pilot study suggested that this was one of the key limitations of the AW4. Since the patient had to manually press the button on the side of the crown to obtain a reading/tracing, there needed to be some awareness on their part that they might be in AF, unless they were in persistent/continuous AF. If the patient had no symptoms then there would not have been an incentive to obtain a reading from the AW4. The AW4 would be better served if it could automatically detect a change in the patient’s rhythm and automatically obtain a reading. However, this is not the case with the current model of the AW4. In addition to the noted drawback of the device, the study noted several limitations which could have affected the accuracy of the results or long-term use-case of this technology for use in the clinic such as: 1) fluid overload and edema following surgery and 2) interference with other medical equipment on or near the patient.

## V. Conclusion

This pilot study represents the first case in evaluating the accuracy of the AW4 to measure heart rate compared to telemetry in a controlled clinical environment. Our results coupled with those previously published by members of this team suggest the need for further validation before wrist-worn heart rate monitors are used in the clinic to inform decision-making protocols.
